# Glycation at the Crossroads of Disease Pathogenesis: Mechanistic Insights and Therapeutic Frontiers

**DOI:** 10.3390/diseases14040137

**Published:** 2026-04-08

**Authors:** Sneha Krishnamoorthi, Rupachandra Saravanakumar, Vivek Kumar

**Affiliations:** Department of Biotechnology, School of Bioengineering, SRM Institute of Science and Technology, Kattankulathur, Chengalpattu 603 203, Tamil Nadu, India; sk7951@srmist.edu.in (S.K.); rupachas@srmist.edu.in (R.S.)

**Keywords:** advanced glycation end products (AGEs), AGE detection methods, AGE–RAGE signalling, controlled glycation, in vitro glycation, oxidative stress and inflammation, protein glycation

## Abstract

Protein glycation is a nonenzymatic modification that links sugar chemistry to molecular aging and chronic disease. Sequential reactions involving Schiff bases, Amadori products, and reactive α dicarbonyl intermediates generate advanced glycation end products (AGEs) that irreversibly alter protein structure and function. AGEs also act as ligands for the receptor for advanced glycation end products (RAGE), initiating oxidative stress, inflammation, and tissue remodeling. This review synthesizes the molecular pathways of AGE formation, their structural diversity, and the biological factors influencing glycation kinetics. Advances in analytical detection methods—including fluorescence spectroscopy, LC–MS/MS, and immunochemical approaches—are highlighted for their role in monitoring AGE accumulation. Particular attention is given to the contribution of glycation to diabetes, cardiovascular disease, neurodegeneration, and cancer, alongside emerging therapeutic strategies to limit AGE formation or block AGE–RAGE signaling. Glycation thus represents a central mechanism in human disease pathogenesis and an emerging therapeutic frontier.

## 1. Introduction

Biological molecules seldom act alone. Within the crowded environment of a cell, proteins, lipids, and nucleic acids are constantly surrounded by sugars and metabolites that test their stability and shape. Among these interactions, glycation stands out as a subtle yet far-reaching reaction, linking the routine chemistry of metabolism to the gradual story of molecular aging. Often described as the Maillard reaction, glycation is a spontaneous nonenzymatic process in which simple sugars or their reactive derivatives attach covalently to amino acid residues such as lysine, arginine, and cysteine [1]. The resulting adducts evolve into Amadori compounds and eventually into advanced glycation end products (AGEs), which alter protein conformation, solubility, and biological activity [2,3]. In this article, ‘glycation products’ refers collectively to early intermediates, including Schiff bases and Amadori products, as well as advanced glycation end products (AGEs), unless otherwise specified. Under conditions of dysregulated glycation, these modifications promote protein misfolding and crosslinked aggregate formation that impair cellular function [4]. These molecular changes underline the contribution of glycation to chronic disorders such as diabetes, cardiovascular disease, neurodegeneration, and cancer [5].

The global impact is profound: type 2 diabetes currently affects nearly 589 million adults and may reach 853 million by 2050, with economic costs projected to surpass USD 2 trillion [6,7]. Alzheimer’s disease, another AGE-associated condition, affects more than 55 million people worldwide and imposes a comparable societal burden [8]. Such figures underscore the urgency of understanding this chemistry. In living systems, glycation proceeds slowly but accelerates with age, becoming a hallmark of molecular aging [2]. Beyond structural damage, AGEs act as signaling molecules by binding to the receptor for advanced glycation end products (RAGE). This interaction triggers oxidative stress, inflammation, and tissue remodeling that contribute to chronic disease [9]. Clinically, glycation serves as both a biomarker and a therapeutic target. Measurements such as glycated hemoglobin and glycated albumin provide indicators of metabolic control [10], while pharmacological and nutritional strategies aim to limit AGE formation, disrupt crosslinks, or block receptor-mediated signaling [11].

**Figure 1:**
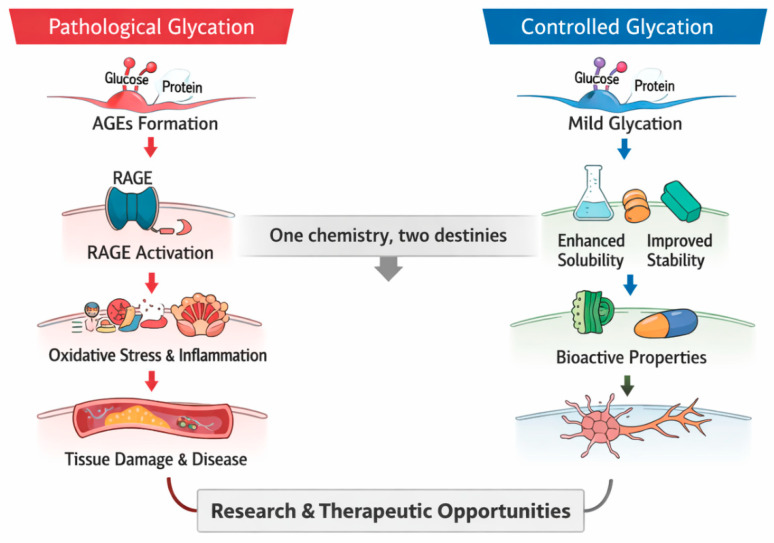
Dual nature of glycation: pathological versus controlled outcomes. Unregulated non-enzymatic glycation in tissues leads to accumulation of advanced glycation end products (AGEs), activation of the receptor for advanced glycation end products (RAGE), and downstream oxidative stress and inflammation associated with chronic disease. In contrast, controlled glycation under laboratory or food-processing conditions can produce limited modifications that improve protein stability, solubility, and functional activity.

Yet glycation is not inherently harmful, and its biological outcome depends on reaction context and extent. Under controlled experimental conditions, mild in vitro glycation can enhance protein stability, solubility, and biological function, creating opportunities in functional foods, nutraceuticals, and therapeutic design [12]. The same chemistry that damages tissues in vivo can, when guided by design, support innovation in science and industry [13]. Figure 1 illustrates this dual nature, contrasting uncontrolled glycation in living systems with deliberate modification in vitro. This apparent duality arises from differences in reaction context, extent, and regulatory control. In vivo, glycation occurs continuously under conditions of metabolic flux, where prolonged exposure to reactive sugars and dicarbonyl intermediates drives progression beyond early reversible intermediates toward irreversible AGE formation. This leads to protein crosslinking, activation of AGE–RAGE signaling, and downstream oxidative and inflammatory responses [14]. In contrast, controlled in vitro glycation is intentionally restricted to early stages of the Maillard reaction—typically limited to Schiff base and Amadori product formation—under defined conditions of temperature, time, and reactant concentration [15]. By preventing progression to advanced glycation end products and associated signaling pathways, this controlled process enables selective modification of protein structure without inducing cytotoxic or inflammatory effects [16]. Thus, the divergent outcomes of glycation reflect differences in reaction control, intermediate stability, and the balance between formation and biological response [14]. This review aims to provide a comprehensive and mechanistically grounded overview of protein glycation, focusing on the pathways of AGE formation, their role in disease pathogenesis, methods for their detection, and emerging therapeutic strategies. In addition, it highlights the context-dependent nature of glycation by contrasting its pathological effects in vivo with its controlled applications in vitro.

## 2. Molecular Mechanism of Protein Glycation

Protein glycosylation is a normal and tightly regulated biological process in which sugar chains are enzymatically attached to proteins during synthesis. This process occurs primarily in the endoplasmic reticulum and Golgi apparatus, where glycosyltransferases catalyze the transfer of sugar moieties to specific amino acid residues. The reactions generate N-linked glycosylation on asparagine or O-linked glycosylation on serine and threonine residues [17]. Enzymatic glycosylation plays an essential role in maintaining correct protein folding, structural stability, subcellular localization, and biological activity [18].

**Figure 2:**
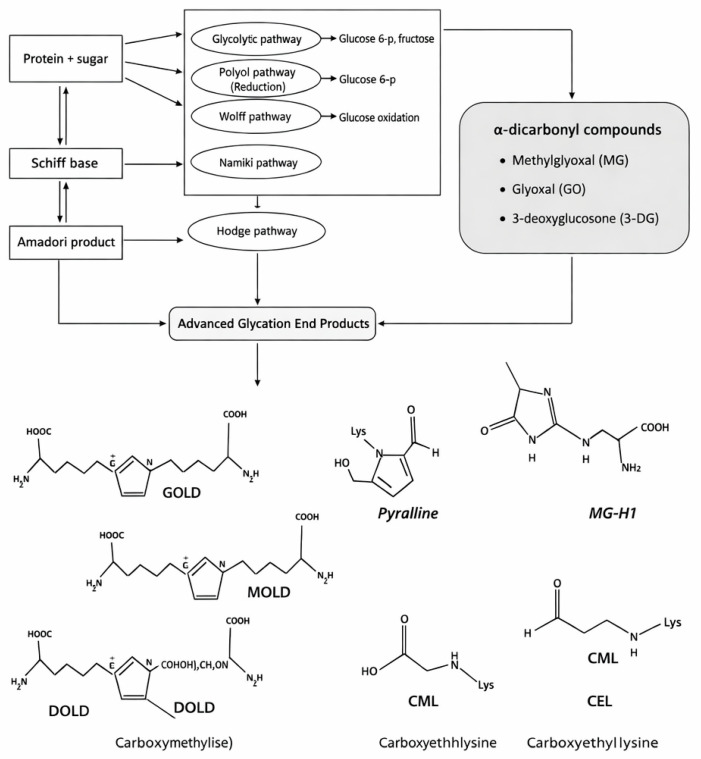
Molecular pathways leading to advanced glycation end products (AGEs). The Maillard reaction proceeds through Schiff base and Amadori product intermediates and contributes to AGE formation via oxidative and degradative routes. α-Dicarbonyl compounds such as methylglyoxal (MG), glyoxal (GO), and 3-deoxyglucosone (3-DG) accelerate AGE production through reactive side chains, yielding characteristic products including CML, CEL, MG-H1, GOLD, MOLD, DOLD, and Pyralline. Adapted from [23] under the Creative Commons Attribution 4.0 License (CC BY 4.0).

In contrast to this enzyme-mediated process, protein glycation occurs through a nonenzymatic mechanism that proceeds via the Maillard pathway. In this reaction, a reducing sugar such as glucose reacts with amino groups of proteins [19]. The reaction predominantly involves the N-terminal α-amino group or the ε-amino group of lysine residues. The initial interaction forms a reversible Schiff base, which can exist in equilibrium between open-chain and cyclic glycosylamine forms [20,21]. Due to its instability, the Schiff base spontaneously rearranges into a more stable ketoamine structure known as the Amadori product [22]. As glycation progresses, the Amadori product undergoes further oxidation, dehydration, and condensation reactions that generate a structurally diverse group of irreversible compounds collectively referred to as advanced glycation end products (AGEs) [2]. The sequential formation of Schiff base and Amadori intermediates and the contribution of α-dicarbonyl compounds to AGE generation are summarized in Figure 2.

In addition to the classical Maillard sequence, protein glycation can proceed through alternative chemical routes. During proteolysis, glycated proteins yield low-molecular-weight fragments and amino acids that retain sugar-derived adducts, referred to as free glycation products [24]. AGEs can also form from reactive carbonyl species (RCS) generated during sugar autoxidation, lipid peroxidation, and glycolytic metabolism [25]. Among these intermediates, methylglyoxal (MG), glyoxal (GO), and 3-deoxyglucosone (3-DG) are the most reactive α-dicarbonyl compounds [26,27]. These molecules react rapidly with nucleophilic side chains, particularly arginine and lysine, and to a lesser extent cysteine residues, producing hydroimidazolone-type AGEs that are prevalent in biological systems subjected to oxidative or metabolic stress [28,29].

A key endogenous defense mechanism against these reactive dicarbonyl intermediates is the glyoxalase system, comprising glyoxalase I (GLO1) and glyoxalase II (GLO2). This pathway catalyzes the detoxification of methylglyoxal and related carbonyl species into less reactive metabolites, thereby limiting intracellular carbonyl stress and subsequent AGE formation. Under physiological conditions, glyoxalase activity maintains cellular proteostasis by preventing excessive glycation [30]. However, in conditions such as hyperglycemia, oxidative stress, and aging, glyoxalase activity may become impaired or saturated, leading to accumulation of reactive dicarbonyls and accelerated AGE generation. This imbalance enhances downstream AGE–RAGE signaling and contributes to the progression of glycation-associated pathologies [30,31].

**Figure 3:**
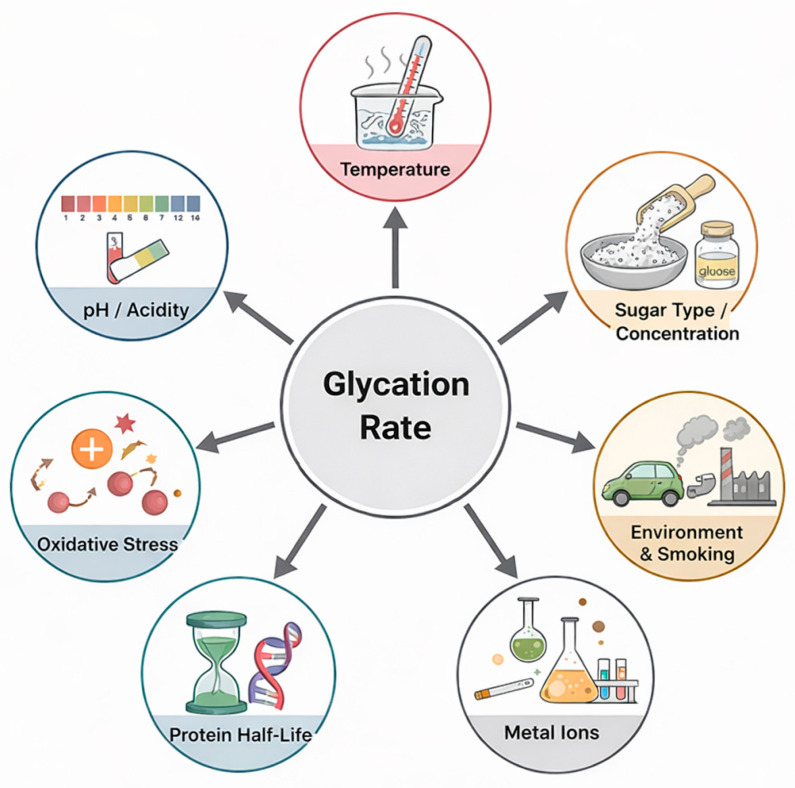
Environmental and molecular factors influencing glycation kinetics. Parameters such as pH, sugar type, temperature, and protein turnover rate determine the extent of glycation and AGE formation under physiological and experimental conditions.

The overall glycation rate and the resulting AGE profile are influenced by environmental and physicochemical conditions. Parameters such as pH, temperature, oxygen concentration, metal ions, and buffer composition determine the reactivity of sugars and the stability of intermediates [32]. The chemical identity of the sugar molecule is another decisive factor. Importantly, not all sugars exhibit the same glycation reactivity. Fructose and other reducing sugars, including ribose, are significantly more reactive than glucose due to their greater propensity to form open-chain intermediates that readily participate in the Maillard reaction [10,15]. As a result, these sugars generate reactive carbonyl intermediates and advanced glycation end products at a faster rate than glucose, thereby accelerating glycation processes under both physiological and experimental conditions [1]. Aldoses generally exhibit higher reactivity than ketoses, and small monosaccharides such as ribose participate in glycation reactions at a faster rate compared with glucose [33]. The biological turnover rate of proteins further contributes to the extent of glycation. Long-lived proteins, such as collagen and crystallins, accumulate irreversible modifications because they remain exposed to reactive sugars and carbonyl compounds for extended durations [34]. Environmental and molecular factors influencing glycation kinetics, including pH, sugar type, temperature, and protein half-life, are summarized in Figure 3. These factors together define the rate and extent of AGE formation under both physiological and experimental conditions, influencing not only tissue vulnerability but also the design of in vitro glycation models.

The sequence of reactions described above defines the molecular progression from early reversible adducts to irreversible AGE formation. This cascade represents the biochemical foundation underlying protein modification associated with chronic hyperglycemia, oxidative stress, and age-related molecular damage.

## 3. Advanced Glycated End Products (AGEs)

**Figure 4:**
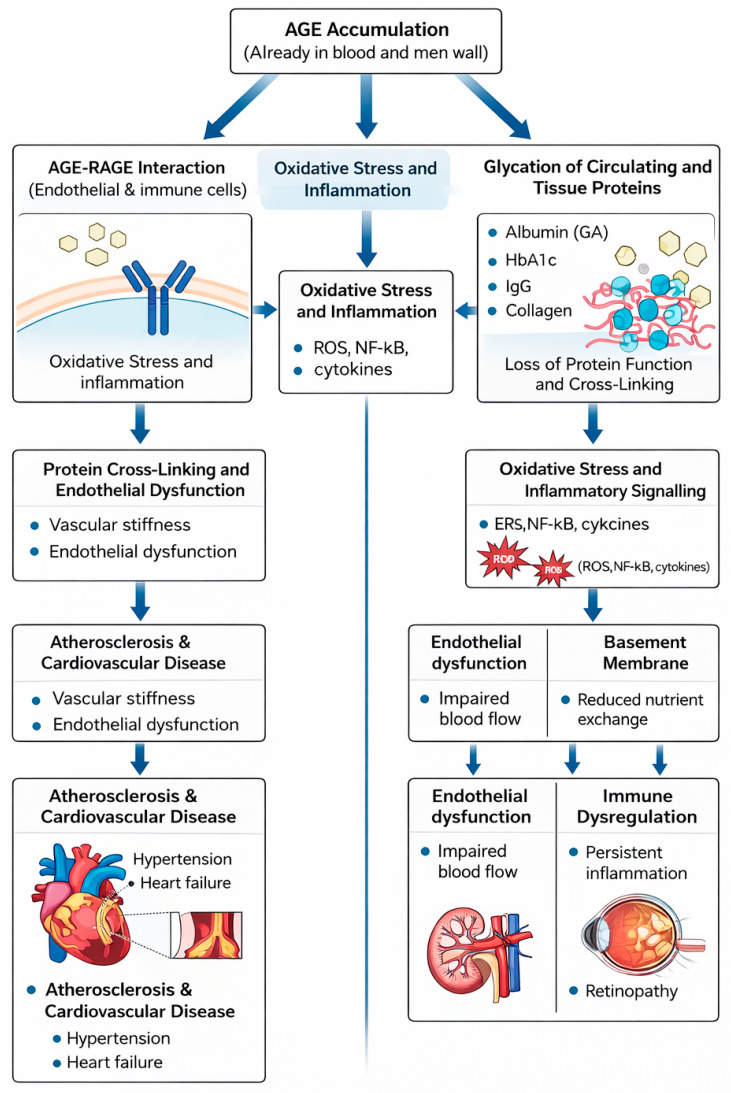
AGE-mediated vascular and metabolic complications. AGE accumulation and RAGE signaling promote oxidative stress, protein crosslinking, and endothelial dysfunction, leading to diabetic and cardiovascular pathologies.

Advanced glycation end products (AGEs) represent the culmination of non-enzymatic glycation, arising from the progressive modification of proteins, lipids, and nucleic acids. These compounds are chemically diverse, encompassing crosslinked structures, fluorescent derivatives, and stable adducts that accumulate irreversibly in long-lived biomolecules [35]. Their formation is driven not only by the classical Maillard pathway but also by reactive carbonyl species such as methylglyoxal and glyoxal, which accelerate AGE generation under conditions of oxidative stress and hyperglycemia [36]. Once formed, AGEs alter the physicochemical properties of proteins, reducing flexibility, impairing solubility, and disrupting enzymatic activity. These structural changes translate into tissue stiffening, loss of elasticity, and impaired cellular communication, thereby linking AGE accumulation to the gradual decline of biological systems. Importantly, the biological significance of AGEs extends beyond structural modification, as they act as bioactive ligands that engage the receptor for advanced glycation end products (RAGE). This interaction initiates intracellular cascades that amplify oxidative stress, sustain inflammation, and promote tissue remodeling [37,38]. Through this receptor-mediated dialogue, AGE chemistry is directly connected to pathophysiological outcomes such as vascular dysfunction, neurodegeneration, and tumor progression, with persistent activation of RAGE signaling now recognized as a defining mechanism in metabolic and age-related diseases [39]. The pathological consequences of AGE accumulation and RAGE activation across vascular and metabolic systems are summarized in Figure 4.

Collectively, these processes illustrate how AGE chemistry bridges molecular reactivity and systemic pathology, providing a biochemical foundation for diagnostic and therapeutic exploration. Because of these dual roles-structural modifiers and signaling molecules-AGEs have also become valuable markers and therapeutic targets. Clinically, glycated hemoglobin (HbA1c) and glycated albumin are widely used to monitor glycaemic control and disease risk. Concurrently, therapeutic strategies aim to limit AGE formation, disrupt AGE-mediated crosslinks, or inhibit AGE–RAGE interactions [40,41]. In addition to proteins, AGEs can also modify lipids and nucleic acids, leading to altered membrane properties and genomic instability, which further contribute to cellular dysfunction and disease progression [42]. Experimental studies have shown that AGE accumulation impairs antioxidant defense systems, thereby amplifying oxidative stress and promoting chronic inflammation [43]. AGE-induced crosslinking of extracellular matrix proteins disrupts normal tissue architecture and mechanical properties, particularly in vascular and connective tissues [44]. These approaches highlight the dual importance of AGEs as both indicators of pathology and targets for intervention, reinforcing their centrality in biomedical research and clinical management [45]. Nanoparticle-based drug delivery systems, including solid lipid nanoparticles, offer promising avenues for targeted delivery of AGE inhibitors and RAGE antagonists [46].

### 3.1. Classification of AGEs

#### 3.1.1. Endogenous and Exogenous AGEs

AGEs are classified into endogenous AGEs, formed within the body, and exogenous AGEs, obtained from external sources such as food and tobacco smoke. Endogenous AGEs are continuously produced during normal sugar metabolism through slow, non-enzymatic glycation reactions in tissues and body fluids. Under physiological conditions, this process is regulated and does not cause significant cellular damage [47]. Based on their chemical precursors, endogenous AGEs can be grouped into distinct types: glyoxal-derived AGEs such as GOLD, CML, and CMA; methylglyoxal-derived AGEs including MG-H1, MOL, CEL, and argpyrimidine; and 3-deoxyglucosone-derived AGEs such as DOLD, pyrraline, and pentosidine, classifications summarized in Table 1. Although structurally diverse, all are generated through sugar-related reactions within cells [48].

Problems arise when endogenous AGEs accumulate excessively, particularly in long-lived proteins such as collagen, which persist for long periods and are especially susceptible to modification [49]. Excessive accumulation promotes oxidative stress and inflammation through protein cross-linking or interaction with cell-surface receptors, leading to altered protein structure and function [50]. These changes activate stress-related signaling pathways and elevate inflammatory mediators, contributing to chronic diseases such as diabetes and cardiovascular disorders [51]. AGE accumulation is also linked to musculoskeletal conditions, including osteoarthritis and rheumatoid arthritis, where increased collagen stiffness reduces bone flexibility and heightens fracture risk, especially in elderly individuals [52,53]. Endogenous AGEs are not easily degraded by common detoxification enzymes and are removed primarily through receptor-mediated uptake and intracellular degradation [54].

In addition to AGEs formed within the body, a substantial fraction arises from exogenous sources, further adding to the total AGE burden. Exogenous AGEs enter mainly through diet and tobacco smoke [55,56]. While small amounts occur naturally in raw animal-derived foods, their concentrations rise sharply during food processing, particularly during high-temperature treatments [50]. Cooking methods such as frying, grilling, roasting, broiling, and searing promote AGE formation via the Maillard reaction between sugars and proteins. Although such thermal processing improves flavor, color, and shelf life, it also accelerates AGE generation—especially in processed foods rich in sugars and proteins [50,56].

Initially, dietary AGEs were thought to be poorly absorbed and of limited biological significance. Subsequent research, however, demonstrated that they can cross the intestinal barrier, enter circulation, and accumulate in tissues, thereby influencing metabolic and inflammatory pathways [57,58]. Among dietary AGEs, CML is one of the most stable and extensively studied compounds and is frequently used as a marker to estimate AGE content in foods [56]. Studies consistently show that meat and animal-based foods, particularly when cooked at high temperatures, contain the highest AGE levels per serving, whereas vegetables, fruits, and whole grains generally contain much lower amounts [59].

#### 3.1.2. Fluorescent and Non-Fluorescent AGEs

The chemical landscape of AGEs extends beyond composition to their optical and structural behavior. Some adducts, known as fluorescent AGEs, emit characteristic fluorescence and readily create covalent crosslinks between neighboring proteins. Compounds such as glyoxal-derived lysine dimer (GOLD), methylglyoxal-derived lysine dimer (MOLD), 3-deoxyglucosone-derived lysine dimer (DOLD), and pentosidine belong to this group. Their yellow–brown chromogenic appearance reflects the molecular rearrangements that accompany advanced stages of the Maillard reaction, and their emission properties allow direct quantification by fluorescence spectroscopy [60]. Through repetitive crosslink formation, these AGEs stiffen extracellular matrices and diminish tissue elasticity, effects that make them both diagnostic signatures and molecular contributors to age- and diabetes-related tissue damage.

Other AGEs remain non-fluorescent, lacking measurable optical emission or significant crosslinking potential, yet their biological influence is far from negligible. Species such as CML, CEL, and pyrraline can disrupt protein conformation, alter charge distribution, and impair receptor interactions [61]. In living tissues, both fluorescent and non-fluorescent AGEs coexist on the same protein scaffolds, producing a chemically heterogeneous network of modifications that mirror the complexity of metabolic history [62]. This coexistence illustrates that glycation is not a single chemical event but a spectrum of molecular outcomes—some visible under light, others silent yet equally consequential.

#### 3.1.3. Relative Cytotoxicity and Glycotoxicity of AGEs

Advanced glycation end products (AGEs) differ in their chemical structure, reactivity, and biological impact; however, accumulating evidence indicates that AGE species can exert cytotoxic and pro-inflammatory effects when present at elevated concentrations or under conditions of impaired detoxification and clearance [63]. Rather than a strict classification into “non-toxic” and “toxic” categories, AGEs are now more appropriately understood within a continuum of glycotoxicity influenced by molecular properties, concentration, and tissue context.

Several well-characterized AGEs, including Nε-(carboxymethyl) lysine (CML), Nε-(carboxyethyl) lysine (CEL), pentosidine, pyrraline, methylglyoxal-derived hydroimidazolone (MG-H1), and cross-linking structures such as GOLD and MOLD, arise through endogenous metabolic processes and carbonyl stress reactions. While some of these AGEs are comparatively less reactive in isolation, their accumulation contributes to oxidative stress, protein dysfunction, and chronic inflammation, particularly through interaction with the receptor for advanced glycation end products (RAGE) [64].

Highly reactive AGE species, particularly those derived from reactive carbonyl compounds such as methylglyoxal and glyceraldehyde, exhibit strong associations with protein crosslinking, mitochondrial dysfunction, and activation of pro-inflammatory signaling pathways, including NF-κB-mediated cytokine release [65]. These processes amplify oxidative stress and disrupt cellular homeostasis, ultimately leading to cellular dysfunction or apoptosis.

The pathological significance of AGEs is therefore closely linked to their cumulative burden rather than the presence of a specific subtype alone. Elevated AGE levels have been implicated in a wide range of lifestyle-related and metabolic diseases, including diabetes mellitus and its vascular complications, cardiovascular disease, NAFLD/NASH, neurodegenerative disorders, and cancer. Their accumulation reflects both metabolic dysregulation and environmental exposure, including dietary intake of AGE-rich foods and reactive sugar metabolites [66].

Collectively, these findings support the concept of AGEs as dose-dependent glycotoxins that contribute to disease progression through sustained oxidative and inflammatory signaling. Representative advanced glycation end products (AGEs), their precursors, and biochemical characteristics are summarized in Table 1.

**Table 1:** Representative advanced glycation end products (AGEs), their precursors, and biochemical characteristics. All listed AGEs have been reported to contribute to glycation-associated cellular dysfunction depending on their accumulation and biological context. Abbreviations: CML, Nε-(Carboxymethyl)lysine; CEL, Nε-(Carboxyethyl)lysine; MGO, methylglyoxal; GO, glyoxal; 3-DG, 3-deoxyglucosone.

AGEs	Chemical Type	Precursor	Fluorescent	Crosslinking	Primary Source	Biological/Pathological Relevance	
CML	Nε-(Carboxymethyl)lysine	GO, Amadori oxidation	No	No	Endogenous & Exogenous	Major biomarker of oxidative glycation; associated with inflammation and vascular damage	[67]
CEL	Nε-(Carboxyethyl)lysine	MGO	No	No	Endogenous & Exogenous	Marker of carbonyl stress; contributes to metabolic and inflammatory dysfunction	[67]
Pentosidine	Crosslinking fluorescent AGE	Ribose, ascorbate, glycoxidation pathways	Yes	Yes	Endogenous & Exogenous	Well-characterized glycotoxin; promotes protein crosslinking, tissue stiffness, and oxidative stress	[68]
MG-H1	Methylglyoxal-derived hydroimidazolone-1	MGO	No	No	Endogenous	Most abundant arginine-derived AGE; implicated in cellular dysfunction and inflammation	[48,69]
GOLD	Glyoxal-derived lysine dimer	GO	Yes	Yes	Endogenous & Exogenous	Strong crosslinking AGE; associated with protein aggregation and oxidative stress	[48,60]
MOLD	Methylglyoxal-derived lysine dimer	MGO	Yes	Yes	Endogenous	Crosslinking AGE contributing to protein rigidity and tissue damage	[67]
DOLD	3-Deoxyglucosone-derived lysine dimer	3-DG	Yes	Yes	Endogenous & Exogenous	Fluorescent crosslinking AGE associated with aging and oxidative stress	[67]
Glucosepane	Crosslinking AGE	Amadori intermediates	No	Yes	Endogenous	Most abundant protein crosslink in human collagen; major contributor to tissue stiffness	[67]

### 3.2. Detection of Advanced Glycation End Products (AGEs)

**Figure 5:**
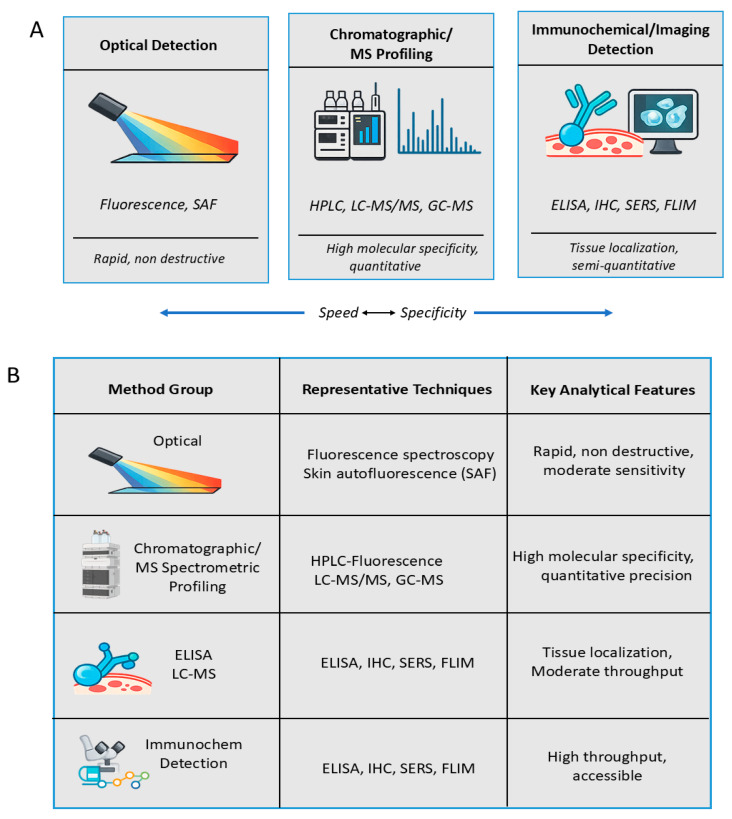
Detection and Analytical Characterization of Advanced Glycation End Products (AGEs). (**A**) Conceptual overview of analytical methods for AGE detection, showing optical, chromatographic/mass spectrometric, and immunochemical/imaging approaches arranged along an axis of analytical depth versus clinical applicability [74,75,76,77,78]. (**B**) Comparative summary of representative techniques highlighting their analytical features and performance characteristics. Representative sources: [72,73,74,75,76,77,78]. The bidirectional arrow indicates the trade-off between analytical speed and molecular specificity across different detection methods.

Detecting AGEs is as much about chemistry as it is about perception. These molecules leave no single signature; instead, they alter how matter absorbs light, scatters electrons, and interact with antibodies. Because AGEs span a wide chemical range—from small, soluble adducts to complex crosslinked proteins—no single method can define them entirely. Modern analytical strategies therefore combine spectroscopic, chromatographic, and immunochemical approaches, each revealing a different dimension of the same molecular landscape [70,71]. The earliest methods exploited the intrinsic fluorescence of certain AGEs such as pentosidine, GOLD, and MOLD, which emit a characteristic signal when excited near 370 nm and detected around 440 nm [72]. This fluorescence index remains a quick, non-destructive marker of AGE accumulation in tissues and body fluids. Yet it captures only the visible subset of the glycation spectrum. Non-fluorescent AGEs like CML and CEL remain invisible to these optical probes, reminding researchers that fluorescence measures presence, not completeness. For deeper resolution, fluorescence assays are now coupled with high-performance liquid chromatography (HPLC) or liquid chromatography–mass spectrometry (LC–MS), which separate and identify AGE species with molecular precision [73]. A conceptual overview of AGE detection strategies, spanning optical, chromatographic, and immunochemical modalities, is shown in Figure 5A,B.

Among all current techniques, LC–MS/MS provides the most comprehensive molecular fingerprinting of AGEs. By resolving compounds based on mass-to-charge ratios and fragmentation patterns, it allows detection of individual species such as CML, CEL, and MG-H1 at nanomolar concentrations in plasma or tissue hydrolysates [74]. Gas chromatography–mass spectrometry (GC–MS), though less commonly used, remains indispensable for volatile AGE derivatives when combined with derivatization. Emerging label-free peptide screening platforms further expand analytical capabilities, offering rapid identification of therapeutic candidates relevant to AGE–RAGE signaling [79]. These approaches reveal not just concentration but structural diversity, connecting analytical data with chemical pathways of formation. Their main limitation lies in accessibility: mass spectrometric profiling demands skilled operators, high instrumentation cost, and standardized reference compound constraints that currently confine it to research laboratories [75].

For clinical and histopathological applications, immunochemical methods remain the most practical. ELISA, Western blotting, and immunohistochemistry employ antibodies targeting specific AGE epitopes such as CML or pentosidine, enabling semi-quantitative detection in plasma, tissues, or cell cultures [76]. In histological sections, immunolabeling reveals AGE accumulation in vascular walls, collagen matrices, and neuronal cells. However, antibody cross-reactivity and structural heterogeneity can confound quantification, necessitating validation through orthogonal analytical techniques. Recent innovations are reshaping how AGEs are visualized in living systems. Surface-enhanced Raman spectroscopy (SERS) and fluorescence lifetime imaging microscopy (FLIM) permit subcellular mapping of AGE deposition, linking chemical state with spatial context [77]. Clinically, skin autofluorescence (SAF) has become a non-invasive surrogate marker, correlating strongly with vascular and metabolic risk in diabetic and aging populations [78]. The next analytical frontier lies in integrative platforms—combining LC–MS quantification with optical or imaging modalities—to translate molecular signatures of glycation into measurable diagnostic indices. Engineered biosystems such as chemically responsive CRISPR-Cas platforms highlight the potential for real-time monitoring and intervention in glycation-linked pathologies [80].

## 4. Role of Glycation in Disease Pathogenesis

Experimental and clinical evidence indicates that glycation contributes to disease development by altering protein structure, triggering oxidative and inflammatory cascades, and modifying extracellular matrix interactions [81]. Through the combined effects of reactive metabolic intermediates, AGE–RAGE signaling, and matrix remodeling, glycation influences fundamental mechanisms that underlie metabolic disorders, cardiovascular disease, neurodegeneration, cancer, and chronic inflammation.

The extent to which glycation contributes to disease is strongly influenced by endogenous detoxification systems, particularly the glyoxalase pathway. Glyoxalase I and II regulate intracellular levels of reactive carbonyl species such as methylglyoxal, thereby modulating AGE formation [82]. Reduced glyoxalase activity, reported in conditions such as diabetes, aging, and neurodegeneration, leads to accumulation of dicarbonyl intermediates, enhanced protein glycation, and amplification of AGE–RAGE–mediated oxidative and inflammatory signaling. Thus, dysfunction of the glyoxalase system represents a critical link between metabolic imbalance and glycation-driven pathology [30].

The downstream biological implications of these molecular mechanisms, previously summarized in Figure 4, manifest across multiple organ systems where chronic AGE accumulation and receptor activation drive tissue-specific dysfunction. The pathological impact of glycation is largely driven by cumulative AGE burden, supporting the concept of AGEs as dose-dependent glycotoxins that amplify oxidative stress and inflammation across multiple organ systems.

Importantly, the pathological effects of AGEs and reactive carbonyl species follow a dose–response relationship governed by both concentration and duration of exposure. Under physiological conditions, low levels of glycation products are continuously generated and efficiently neutralized by endogenous detoxification systems, including the glyoxalase pathway and antioxidant defenses [83]. However, when the rate of formation exceeds the capacity for detoxification—such as during sustained hyperglycemia, oxidative stress, or increased dietary AGE intake—reactive dicarbonyl compounds like methylglyoxal accumulate, leading to carbonyl stress. This transition results in a nonlinear escalation of AGE formation, enhanced protein modification, and intensified activation of AGE–RAGE–mediated signaling pathways [83]. Consequently, chronic exposure to elevated AGE levels drives progressive tissue damage, highlighting that both the magnitude and persistence of glycation are critical determinants of disease progression [42].

### 4.1. Glycation in Cardiovascular Disease

In cardiovascular diseases (CVDs), non-enzymatic glycation promotes the accumulation of AGEs in both circulating and tissue proteins, including long-lived extracellular matrix components such as collagen and elastin, as well as intracellular proteins in vascular and cardiac cells [84]. Glycation of collagen and elastin induces intermolecular cross-linking that increases vascular and myocardial stiffness, reduces arterial compliance, and contributes to hypertension, diastolic dysfunction, and heart failure [85].

Simultaneously, AGE interaction with the receptor for advanced glycation end products (RAGE) on endothelial cells, smooth muscle cells, and immune cells initiates oxidative stress, inflammatory signaling, and endothelial dysfunction, accelerating atherosclerotic progression [86]. Several circulating proteins undergo glycation in CVDs, including hemoglobin (HbA1c), serum albumin (fructosamine), and apolipoprotein B in low-density lipoprotein particles (ApoB-G), each reflecting cumulative glycemic exposure over different time scales [87]. Glycation of ApoB modifies lysine residues near the LDL receptor–binding domain, reducing receptor-mediated LDL clearance and prolonging its circulation in plasma. Consequently, glycated LDL is preferentially taken up by macrophages through scavenger pathways, enhancing foam-cell formation and accelerating atherosclerotic plaque development [88]. In addition, glycated LDL is more prone to oxidative modification, increases monocyte adhesion, promotes cholesteryl ester accumulation within macrophages, and enhances platelet aggregation—amplifying vascular injury and elevating thrombotic risk [89].

Epidemiological studies consistently show that elevated levels of HbA1c, fructosamine, and ApoB-G correlate with atherosclerotic lesions, myocardial infarction, and cardiovascular mortality, even in non-diabetic populations. These findings underscore protein glycation as both a mechanistic driver and a clinically useful biomarker for early cardiovascular risk assessment [90].

### 4.2. Glycation in Neurodegenerative Disorders

Neurodegenerative disorders such as Alzheimer’s disease and Parkinson’s disease arise from complex and multifactorial mechanisms, including protein misfolding, impaired proteostasis, genetic susceptibility, and mitochondrial dysfunction [91]. In this review, these conditions are discussed specifically in the context of glycation, which acts as a modifying factor that influences protein aggregation, oxidative stress, and inflammatory signaling rather than as a primary initiating cause.

Glycation-induced molecular stress extends into the central nervous system, where neurons are highly vulnerable because of their elevated energy demand and limited antioxidant capacity. The brain’s lipid-rich environment and long-lived proteins make it particularly prone to carbonyl modification [92]. Accumulating AGEs disrupt protein structure, impair mitochondrial function, and activate glial inflammatory pathways. These changes weaken synaptic signaling and accelerate neurodegeneration. Increasing evidence indicates that glycation contributes to the progression of disorders such as Alzheimer’s disease, Parkinson’s disease [93], and other dementias by linking metabolic imbalance to progressive neuronal injury.

#### 4.2.1. Alzheimer’s Disease (AD)

Alzheimer’s disease (AD) is a progressive neurodegenerative disorder characterized by gradual cognitive decline and neuronal loss, predominantly affecting the hippocampus and cortical regions of the brain [94]. Although most cases arise sporadically, genetic variants including the ε4 allele of apolipoprotein E (ApoE4) and mutations in amyloid precursor protein (APP) substantially elevate disease risk [95]. Protein glycation has emerged as a major contributor to AD pathology, altering the folding and stability of neuronal proteins and promoting their abnormal aggregation. During glycation, normal α-helical conformations progressively convert into β-sheet-rich assemblies that favor amyloid fibril formation [96]. Advanced glycation end products (AGEs), generated through reactions between reactive carbonyl compounds and lysine or arginine residues [97], are found at significantly higher concentrations within β-amyloid plaques and neurofibrillary tangles (NFTs) in AD brains than in age-matched controls [98]. Their presence within these hallmark lesions suggests an active role in modulating disease progression rather than passive accumulation with aging. While Alzheimer’s disease is primarily driven by amyloid-β accumulation, tau pathology, and proteostatic imbalance [99], glycation is discussed here as a contributing mechanism that exacerbates protein aggregation and downstream oxidative and inflammatory pathways.

Beyond structural modification, AGEs engage the receptor for advanced glycation end products (RAGE), a transmembrane receptor abundantly expressed in neurons, astrocytes, and endothelial cells. RAGE also recognizes β-amyloid (Aβ), and this dual binding promotes receptor clustering and intracellular signaling that amplifies oxidative stress through NF-κB and MAPK activation [100]. The sequence of AGE–RAGE–mediated oxidative and inflammatory events within neural tissues is illustrated in Figure 4, which depicts the converging pathways linking glycation chemistry to neuronal toxicity and neuroinflammation.

The connection between diabetes mellitus and AD provides further evidence of glycation’s central role in neurodegeneration. Individuals with diabetes exhibit increased accumulation of AGEs and elevated RAGE expression in the brain, particularly within microglia and cerebrovascular endothelium. Those affected by both diabetes and AD display more extensive amyloid plaque deposition, greater tau pathology, and heightened inflammatory activity compared with patients suffering from AD alone [101]. Glycation enhances Aβ cross-linking, thereby accelerating fibril growth and plaque expansion. Glycated Aβ is markedly more cytotoxic than its unmodified form, as it triggers RAGE-dependent activation of glycogen synthase kinase-3 (GSK-3) and other kinases that impair neuronal signaling. Experimental inhibition of glycation or blockade of RAGE signaling has been shown to reduce neuronal injury and improve cognitive outcomes in animal models [102].

Glycation also plays a crucial role in tau pathology. AGEs colocalize with hyperphosphorylated tau within neurofibrillary tangles, where they induce oxidative stress and disrupt microtubule stability [103]. AGE–RAGE signaling promotes GSK-3 activation, leading to abnormal tau phosphorylation and synaptic impairment. Reactive dicarbonyl species such as methylglyoxal further amplify both Aβ and tau glycation, reinforcing a cycle of proteotoxic stress [104]. In advanced AD, diminished activity of detoxifying enzymes such as glyoxalase I leads to increased accumulation of reactive dicarbonyl species, particularly methylglyoxal. This elevation in carbonyl stress enhances glycation of neuronal proteins and promotes AGE formation, which in turn amplifies AGE–RAGE–mediated oxidative and inflammatory signaling. Concurrently, metabolites such as glyceraldehyde and methylglyoxal disrupt neuronal metabolism, establishing a self-sustaining loop of oxidative injury, inflammation, and neuronal apoptosis that underlies progressive cognitive decline in Alzheimer’s disease [105,106].

#### 4.2.2. Parkinson’s Disease (PD)

Parkinson’s disease (PD) is a chronic neurodegenerative disorder characterized primarily by motor impairment, bradykinesia, rigidity, and resting tremor resulting from the progressive loss of dopaminergic neurons in the substantia nigra pars compacta [107]. Although its exact cause remains multifactorial, a combination of genetic predisposition and environmental exposure contributes to disease onset and progression. Among the molecular hallmarks of PD is the misfolding and aggregation of α-synuclein, a small, intrinsically disordered protein that forms intracellular inclusions known as Lewy bodies [108]. This aggregation process is driven by multiple factors, including oxidative stress, mitochondrial dysfunction, impaired proteostasis, and genetic mutations (e.g., SNCA, LRRK2), which collectively influence α-synuclein misfolding and accumulation [109].

Under physiological conditions, α-synuclein interacts loosely with lipid membranes and synaptic vesicles. During glycation, reactive carbonyl species modify multiple lysine residues, inducing conformational transitions that promote β-sheet formation and self-association [110]. Glycated α-synuclein exhibits enhanced aggregation kinetics, accelerating the nucleation of oligomers and fibrils observed in PD brains [111]. Post-mortem analyses consistently reveal elevated AGE levels co-localized with α-synuclein deposits in the substantia nigra, indicating that glycation actively contributes to Lewy body pathology [112]. The biochemical interplay between AGE accumulation, α-synuclein misfolding, and oxidative stress is schematically illustrated in Figure 4, which summarizes the common AGE–RAGE–driven mechanisms underlying neuronal injury in degenerative disorders. Glycation of α-synuclein alters its physiological behavior in several damaging ways. The modified protein forms soluble oligomers that are highly neurotoxic compared with mature fibrils [113]. These oligomers disrupt neuronal membranes by forming pore-like structures, disturbing ion gradients, and impairing cellular homeostasis [114]. Glycated α-synuclein also enhances mitochondrial reactive-oxygen-species production, intensifying oxidative stress and energy depletion. Poor proteasomal clearance allows glycated α-synuclein to accumulate, triggering proteostatic stress and secondary autophagy [115]. Sustained exposure to glycated α-synuclein further stimulates microglial activation, resulting in chronic neuroinflammation and progressive dopaminergic cell loss [115].

A critical link between glycation and neuroinflammation is mediated through the receptor for advanced glycation end products (RAGE). Glycated α-synuclein binds to RAGE on neurons and glia, activating NF-κB–dependent transcription of pro-inflammatory cytokines [116]. This interaction amplifies RAGE expression itself, creating a self-perpetuating feedback loop that maintains oxidative and inflammatory stress. Over time, these processes converge to drive the selective vulnerability of substantia nigra neurons and the clinical progression of PD.

Collectively, glycation represents an important contributing mechanism in Parkinson’s disease, promoting toxic α-synuclein oligomerization, impairing protein-clearance systems, sustaining oxidative imbalance, and amplifying RAGE-mediated neuroinflammation [116]. Understanding this biochemical convergence offers a promising framework for developing therapeutic strategies aimed at carbonyl stress reduction and modulation of AGE–RAGE signaling.

#### 4.2.3. Amyotrophic Lateral Sclerosis (ALS) and Familial Amyloid Polyneuropathy (FAP)

Glycation also contributes to neurodegenerative diseases beyond Parkinson’s, particularly amyotrophic lateral sclerosis (ALS) and familial amyloid polyneuropathy (FAP) [117]. In ALS, neurofilament proteins are susceptible to modification at lysine-rich segments, while superoxide dismutase-1 (SOD1) undergoes glycation at Lys122 and Lys128 [118]. These molecular alterations promote cross-linking and aggregation, weakening the cytoskeletal integrity of motor neurons and accelerating their degeneration. Reduced concentrations of soluble RAGE in circulation exacerbate the inflammatory cascade, allowing glycated protein aggregates to persist within neural tissues [119]. Early immune responses may transiently mitigate damage, but persistent oxidative and inflammatory stress ultimately overwhelms compensatory defenses [120].

In FAP, glycation destabilizes the transthyretin (TTR) tetramer, favoring misfolded conformers that readily assemble into amyloid fibrils [121]. Elevated levels of methylglyoxal-derived AGEs detected in FAP patients indicate that carbonyl and metabolic stress may accelerate amyloidogenesis [122]. Glycated TTR exhibits enhanced affinity for RAGE, triggering oxidative stress and NF-κB-dependent transcription of inflammatory mediators such as IL-1β and TNF-α [123]. Together, these events establish a mechanistic link between carbonyl stress, protein misfolding, and chronic inflammation in FAP progression [124].

### 4.3. Glycation in Inflammation and Diabetes

In hyperglycemia, excess glucose reacts with amino groups of proteins such as albumin, initiating a reversible Schiff base that subsequently rearranges into a more stable Amadori product. These intermediates undergo oxidative transformation to form reactive dicarbonyl species, which further react to yield advanced glycation end products (AGEs). The progressive glycation of proteins modifies their structural conformation, charge distribution, and binding characteristics, reducing enzymatic activity and promoting aggregation and cross-linking [125]. These structural distortions facilitate AGE interaction with RAGE, activating intracellular cascades that elevate inflammatory mediators including TNF-α, IL-6, and IL-8 [126]. The sequence of molecular events leading from AGE formation to RAGE-mediated oxidative and inflammatory signaling is summarized in Figure 4.

Among circulating proteins, glycated albumin (GA) plays a particularly active role in inflammation. By engaging PKC and NADPH oxidase pathways, GA enhances oxidative stress and upregulates adhesion molecules, leading to immune-cell recruitment in vascular, renal, and retinal tissues. Owing to its shorter half-life, GA reflects glycaemic control over a period of two to three weeks, making it an effective intermediate-term biomarker in clinical contexts where HbA1c may be unreliable, such as pregnancy or dialysis [127].

Sustained hyperglycemia intensifies this process. In addition to glucose, dietary sugars such as fructose—commonly present in sweeteners like high-fructose corn syrup—can significantly contribute to glycation [128]. Despite producing a lower glycemic response, fructose has been shown to exhibit higher intrinsic glycation reactivity and generates advanced glycation end products more rapidly than glucose, due in part to its greater tendency to form reactive carbonyl intermediates [129]. Consequently, high dietary intake of such sugars may enhance AGE accumulation and carbonyl stress independently of blood glucose levels, highlighting their potential role in metabolic and inflammatory disorders. At the molecular level, proteins such as serum albumin, hemoglobin (HbA1c), and immunoglobulin G (IgG) accumulate glycation adducts that trigger RAGE activation on endothelial and immune cells. This receptor engagement amplifies oxidative stress and activates NF-κB and MAPK signaling, perpetuating cytokine release and systemic inflammation [130]. The resulting molecular feedback contributes to metabolic dysregulation, vascular stiffening, and reduced immune competence, highlighting glycation as a key contributing mechanism in diabetic complications.

Glycation of long-lived extracellular proteins such as collagen—whose half-life ranges from several years to decades—further compounds these effects [84]. Cross-linking of glycated collagen thickens basement membranes, decreases elasticity, and narrows microvascular lumens, contributing to diabetic nephropathy and retinopathy [131]. Simultaneously, glycation of IgG interferes with Fc-receptor interactions, weakening antibody-dependent immune responses while enhancing inflammation through AGE–RAGE activation. Elevated levels of glycated IgG have therefore been proposed as a marker of long-term glycaemic exposure and immune alteration in diabetes [132].

In contrast, circulating proteins such as serum albumin have a relatively short turnover time of approximately 4–5 weeks, which corresponds to a glycation assessment window of about 2–3 weeks. This rapid turnover limits prolonged accumulation of advanced glycation end products, making albumin a sensitive marker of short-term glycation status [133]. This distinction highlights how both protein lifespan and concentration influence the biological impact of glycation.

Chronic glycation-driven inflammation extends beyond metabolic dysfunction and has been increasingly implicated in tumor initiation and progression through sustained oxidative stress, angiogenic signaling, and immune modulation—thereby providing a biochemical link to glycation’s emerging role in cancer.

### 4.4. Glycation and Cancer Progression

Glycation plays an important role in cancer development and progression, mainly through the formation and accumulation of advanced glycation end products (AGEs). AGE levels increase markedly under conditions such as poor diet, obesity, physical inactivity, and aging [134]. In vivo, AGEs accumulate gradually within tissues and organs, and insufficient clearance leads to protein damage, altered signaling, oxidative stress, and genomic instability. Increasing evidence indicates that this accumulation creates a biological environment that favors tumor initiation, growth, and aggressiveness [135]. Lifestyle factors such as smoking, alcohol consumption, and sedentary behavior further increase AGE burden. Elevated circulating and tissue AGE levels have been reported in several human cancers, including breast, prostate, colon, and pancreatic tumors, where higher AGE content correlates with aggressive phenotypes and reduced survival [135].

A central mechanism linking glycation to cancer involves AGE interaction with its cellular receptor, the receptor for advanced glycation end products (RAGE). RAGE is frequently overexpressed in tumors, and AGE–RAGE engagement activates multiple pro-inflammatory and pro-tumorigenic pathways [136]. Downstream signaling stimulates transcription factors such as NF-κB, STAT3, and HIF-1α, increasing the expression of cytokines including IL-1β, IL-6, and TNF-α. This cascade promotes immune-cell recruitment to the tumor microenvironment, elevates reactive oxygen species (ROS), and sustains chronic inflammation—conditions that support cancer cell survival, proliferation, invasion, and metastasis [137]. The central molecular events linking AGE–RAGE activation with tumor-promoting inflammation and oxidative stress are illustrated in Figure 4. Oxidative stress forms a critical link between glycation and malignancy. AGE formation itself produces reactive intermediates that elevate ROS levels, while excessive ROS further accelerates the generation of AGE precursors such as methylglyoxal, creating a self-amplifying cycle of oxidative injury and glycation [137]. This persistent redox imbalance fosters genetic and epigenetic alterations, disrupts normal regulatory signaling, and contributes directly to tumor initiation and progression. Chronic inflammation emerging from this process is a recognized hallmark of cancer and is strongly influenced by metabolic and lifestyle-associated glycation [137].

Race-specific differences in AGE accumulation and inflammatory responses have also been observed in cancer. African American patients frequently show higher circulating and tumor AGE levels accompanied by increased markers of inflammation and oxidative stress [138]. These biological variations are closely associated with socioeconomic and lifestyle factors, such as poor diet, limited access to healthy foods, and reduced physical activity—which contribute to elevated exogenous AGE exposure. Consequently, glycation represents a key biological link connecting lifestyle risk factors to tumor behavior, disease aggressiveness, and clinical outcomes, contributing to observed disparities in cancer progression [138]. Elevated dietary AGE intake further accelerates tissue accumulation. In the tumor milieu, these AGEs bind to RAGE on both normal and malignant cells, triggering inflammatory transcriptional programs and promoting cytokine release, immune-cell recruitment, and enhanced ROS generation [139,140]. The oxidative and inflammatory cycles reinforce one another, sustaining cellular stress that supports tumor growth and invasion.

Collectively, uncontrolled in vivo AGE accumulation represents a pathogenic mechanism in cancer. Through persistent AGE–RAGE-mediated inflammation and oxidative stress, glycation functions as a metabolic and lifestyle-driven process that fosters tumor initiation, progression, and poor clinical outcomes by maintaining a pro-tumorigenic microenvironment [140].

## 5. Therapeutic Strategies Targeting Glycation

Because glycation contributes to the onset and progression of numerous chronic diseases, several therapeutic strategies have been explored to limit AGE formation, disrupt established cross-links, or inhibit AGE–RAGE signaling. These include pharmacological agents, natural compounds, and lifestyle modifications that collectively aim to reduce the glycation burden and mitigate its biological consequences.

### 5.1. AGE Formation Inhibitors

Agents that inhibit AGE formation act mainly by trapping reactive carbonyl intermediates or interfering with the early Maillard reaction. Aminoguanidine remains one of the best-characterized carbonyl scavengers, reacting with reactive dicarbonyls to slow AGE accumulation in tissues [141]. Pyridoxamine, a vitamin B6 derivative, captures intermediates generated during glucose and lipid oxidation, thus interrupting downstream glycation. Thiamine and its lipid-soluble analog benfotiamine enhance the transketolase pathway, diverting glucose metabolism away from AGE precursor formation [142]. Certain antidiabetic drugs, including metformin and buformin, exert additional anti-glycation effects by reducing oxidative stress and decreasing reactive carbonyl intermediates. Non-steroidal agents such as diclofenac have also demonstrated the ability to hinder early sugar–protein interactions [143]. Collectively, these compounds exemplify strategies that target the earliest stages of the glycation cascade to prevent long-term molecular and tissue damage.

### 5.2. AGE Cross-Link Breakers

While formation inhibitors prevent new AGEs, cross-link breakers aim to reverse structural rigidity caused by existing AGE-mediated protein cross-links. These compounds act primarily on long-lived extracellular proteins, such as collagen and elastin, that accumulate cross-links with age [144]. Alagebrium chloride (ALT-711), one of the most extensively studied agents, disrupts α-diketone–based cross-links, improving arterial compliance and tissue elasticity in experimental and clinical models [145]. Phenacylthiazolium bromide (PTB) exhibits similar activity, cleaving preformed AGE bridges and restoring protein flexibility. Although clinical translation remains limited, these agents provide proof of concept that structural AGE damage may be partially reversible [146].

### 5.3. RAGE Antagonists

Blocking the AGE–RAGE axis represents another promising therapeutic avenue. FPS-ZM1 is a high-affinity RAGE inhibitor that prevents ligand binding and suppresses downstream NF-κB and MAPK activation. Azeliragon similarly antagonizes RAGE signaling and has demonstrated potential in reducing neuroinflammatory injury [147]. Endogenous soluble RAGE (sRAGE) acts as a natural decoy, binding circulating AGEs and preventing engagement of membrane-bound receptors. Recombinant sRAGE administration and anti-RAGE antibodies are being evaluated as therapeutic tools to neutralize receptor-mediated inflammation [147]. Through these mechanisms, RAGE antagonists reduce oxidative and inflammatory cascades, interrupting a key signaling loop in glycation-driven pathophysiology.

### 5.4. Natural Anti-Glycation Compounds

Several naturally occurring molecules exhibit both anti-glycation and antioxidant properties. Polyphenols such as curcumin, resveratrol, and epigallocatechin gallate (EGCG) inhibit early glycation reactions, suppress oxidative stress, and downregulate inflammatory mediators associated with AGE signaling [148]. The dipeptide carnosine prevents cross-link formation by sequestering reactive carbonyl species and chelating catalytic metals that promote oxidation [149]. Nutrients such as thiamine and pyridoxamine further protect by supporting metabolic detoxification of reactive intermediates. Additionally, endogenous defense systems—including fructosamine-3-kinase (FN3K), aldose reductase, and the glyoxalase pathway—play intrinsic roles in detoxifying Amadori products and reactive dicarbonyls, maintaining proteostasis and cellular redox balance [150].

In addition to well-characterized compounds, a wide range of natural products derived from terrestrial and marine sources have been reported to exhibit anti-glycation activity. Additional phytochemical classes, including flavonoids (e.g., quercetin, catechins), phenolic acids (e.g., chlorogenic acid, ferulic acid), and tannins, have been shown to inhibit AGE formation through mechanisms including reactive carbonyl scavenging, metal chelation, and attenuation of oxidative stress [151]. Plant-derived extracts such as green tea and cinnamon have also been extensively investigated for their capacity to modulate glycation pathways [152]. Furthermore, marine-derived compounds, including phlorotannins from brown algae and sulfated polysaccharides from seaweeds, have been increasingly investigated for their ability to modulate protein glycation and associated oxidative processes [153]. These findings highlight the potential of natural products as multifunctional modulators of glycation and carbonyl stress.

### 5.5. Lifestyle and Dietary Interventions

Lifestyle and dietary habits have a strong influence on the formation and accumulation of AGEs in the body. AGEs are produced naturally during normal metabolism and are also obtained from the diet. Foods cooked at high temperatures, such as frying, grilling, roasting, and broiling, contain high levels of AGEs, whereas cooking methods such as boiling, steaming, and slow cooking significantly reduce AGE formation [154]. Diets rich in processed foods, processed carbohydrates, high-fat animal products, and high-fructose corn syrup further increase glycation, while diets containing more fruits, vegetables, and minimally processed foods contribute lower AGE levels [155].

Human dietary intervention studies indicate that reducing dietary AGE intake can lower circulating AGE concentrations and improve insulin sensitivity, suggesting a protective metabolic effect [156]. In addition to diet, lifestyle factors also play an important role. Smoking promotes the formation and accumulation of AGEs in plasma and tissues, contributing to tissue damage and inflammation [157]. Regular moderate physical activity has been associated with reduced circulating AGEs and beneficial changes in soluble RAGE levels in several human studies, although results vary across populations [158,159]. Overall, maintaining normal blood glucose levels, avoiding smoking, engaging in regular exercise, and adopting low-AGE dietary choices and cooking practices are effective non-pharmacological strategies to reduce glycation and limit AGE-related metabolic and inflammatory effects.

## 6. In Vitro Glycation and Its Beneficial Applications

**Figure 6:**
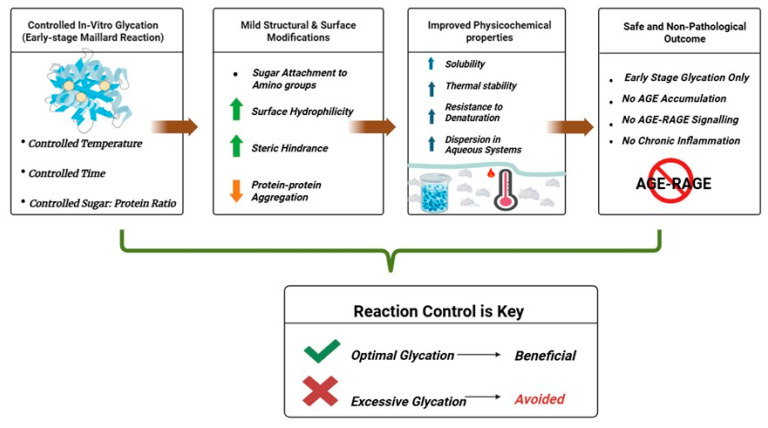
In vitro glycation and its beneficial biochemical applications. Controlled glycation reactions can enhance the structural and functional properties of proteins when precisely regulated under laboratory conditions. The schematic contrasts uncontrolled in vivo glycation, which leads to protein cross-linking, oxidative stress, and disease, with mild in vitro glycation, which improves protein solubility, stability, and bioactivity. Such deliberate modifications are increasingly used in biotechnology, food science, and therapeutic formulation, demonstrating how a reaction that drives pathology in vivo can serve as a constructive biochemical tool when carefully optimized.

The beneficial effects of controlled and mild glycation in stabilizing proteins, improving solubility, and enhancing functional bioactivity are illustrated in Figure 6. These schematics contrast pathological glycation with intentionally modulated in vitro glycation, emphasizing its biotechnological potential when properly regulated.

### 6.1. Concept of In Vitro Glycation

In vitro glycation refers to the deliberate, non-enzymatic attachment of reducing sugars to proteins under strictly defined experimental conditions. Unlike the slow, spontaneous reactions that occur in vivo, laboratory glycation is typically accelerated through elevated sugar concentrations, controlled temperature, and optimized incubation times, producing early intermediates such as Schiff bases and Amadori products within a manageable timescale [160]. Comparative analyses show that in vitro models generate a broader and more heterogeneous modification profile than physiological glycation. For example, experiments on α-crystallin reveal that in vitro glycation leads to a greater number of Amadori products and distinct lysine modifications compared with those formed in human tissues [161,162]. These models are thus designed mainly to elucidate reaction chemistry, kinetics, and inhibition, rather than to reproduce the complex regulatory environment of living systems.

Recent work confirms that accelerated glycation models are indispensable for mechanistic and analytical insight, provided that their outcomes are interpreted within their experimental boundaries [163]. The characterization of in vitro glycation and its modulation relies on a range of analytical techniques that enable detailed assessment of protein modification and AGE formation. Electrophoretic approaches, particularly SDS–PAGE, are commonly used to evaluate glycation-induced changes in protein mobility, aggregation, and crosslinking [164]. Fluorescence-based assays provide sensitive detection of AGE formation, especially for intrinsically fluorescent AGEs such as pentosidine, allowing real-time monitoring of glycation kinetics [81]. Complementary techniques, including spectroscopic and chromatographic analyses, further support structural and compositional evaluation of glycated proteins. These analytical platforms are also widely employed to assess the efficacy of anti-glycation compounds and deglycating enzymes, such as fructosyl peptide oxidases, by quantifying reductions in glycation markers or restoration of native protein characteristics [165].

### 6.2. Beneficial Functional Outcomes of Controlled In Vitro Glycation

Controlled in vitro glycation has emerged as a rational approach to enhance protein functionality and resilience through mild, well-regulated structural modification during the early Maillard stages. When temperature, moisture, and sugar-to-protein ratios are finely tuned, reducing sugars form stable conjugates with protein amino groups without progressing toward advanced glycation end-products (AGEs) [166]. This controlled reaction increases surface hydrophilicity, introduces steric spacing, and limits intermolecular aggregation, thereby improving solubility and dispersion in aqueous systems [15]. Structural adjustments promote thermal stability and improve emulsifying and foaming properties by optimizing interfacial interactions. In addition, surface charge redistribution and subtle conformational flexibility enhance the protein’s adaptability to processing environments.

Beyond physicochemical improvements, controlled glycation enhances antioxidant activity, as early Maillard-derived intermediates can scavenge reactive oxygen species and stabilize functional peptides [15]. However, these benefits arise only within a narrow reaction window; excessive glycation can mask essential amino acids and compromise digestibility. Importantly, since this process occurs outside living systems and halts before AGE formation, it avoids the inflammatory AGE–RAGE signaling associated with pathological glycation. The principle of achieving beneficial outcomes through controlled glycation, as previously illustrated in Figure 6, underscores the conceptual transition from glycation as a source of damage to glycation as a tool for molecular innovation [167].

### 6.3. Functional and Biomedical Examples of Controlled In Vitro Glycation

Experiments on proteins such as nisin, lysozyme, whey proteins, bovine serum albumin, ovalbumin, and casein demonstrate that mild in vitro glycation can enhance solubility, heat resistance, and antioxidant potential while introducing new functional properties. Glycated nisin, for example, exhibits enhanced anticancer activity, inducing apoptosis in breast cancer cells at lower concentrations than the untreated form, suggesting strong therapeutic potential. Glycated lysozyme and whey proteins also show improved antimicrobial strength, greater oxidative stability, and increased tolerance to environmental stress [168,169,170,171,172]. These findings support the concept illustrated in Figure 6, where controlled glycation converts a potentially harmful chemical reaction into a functional biological advantage. Equally important is the safety of this approach. Because reactions are restricted to early glycation stages and performed entirely in vitro, the resulting conjugates remain free from AGE–RAGE–mediated cytotoxicity. The modified proteins retain biochemical stability and biological activity, distinguishing controlled glycation as a refined biochemical adjustment rather than a mimic of disease chemistry.

Together, these studies establish in vitro glycation as a flexible and purposeful platform that links basic chemistry to applied biotechnology. It no longer serves merely as a model of damage but as a powerful tool for designing stable, active, and functional biomolecules that combine molecular precision with translational utility.

## 7. Conclusions

Glycation represents a biochemical crossroad where the same chemistry can either drive pathology or enable innovation, depending on context and control. In vivo, uncontrolled glycation leads to the accumulation of advanced glycation end products (AGEs) that distort protein structure, stiffen extracellular matrices, and activate AGE–RAGE signaling cascades, fuelling oxidative stress, inflammation, and chronic disorders including diabetes, cardiovascular disease, neurodegeneration, cancer, and musculoskeletal decline. Modern detection strategies—spanning fluorescence spectroscopy, immunoassays, and LC–MS/MS profiling—have revealed the chemical diversity of AGEs and established them as both mechanistic drivers and clinically valuable biomarkers. Conversely, controlled in vitro glycation has emerged as a purposeful tool, where mild, early-stage modifications enhance protein solubility, stability, antioxidant potential, and bioactivity without inducing cytotoxicity, as demonstrated in model proteins such as nisin, lysozyme, and whey proteins. This duality reframes glycation not as an inevitable molecular hazard but as a controllable biochemical principle that bridges fundamental chemistry with applied biotechnology, offering translational opportunities across medicine, nutrition, and materials science. Recognizing and exploiting this distinction is central to future research aimed at converting glycation from a marker of pathology into a deliberate means of molecular refinement and biomedical advancement.

## Data Availability

No new experimental data were generated during the preparation of this review. All data are taken from the cited literature. All figures are made from scratch by authors.
